# Measurement of Vital Signs by Lifelight Software in Comparison to Standard of Care Multisite Development (VISION-MD): Protocol for an Observational Study

**DOI:** 10.2196/41533

**Published:** 2023-01-11

**Authors:** Laura Wiffen, Thomas Brown, Annika Brogaard Maczka, Melissa Kapoor, Laurence Pearce, Milan Chauhan, Anoop J Chauhan, Manish Saxena, Lifelight Trials Group

**Affiliations:** 1 Department of Research and Innovation Queen Alexandra Hospital Portsmouth Hospitals University NHS Trust Portsmouth United Kingdom; 2 Mind Over Matter MedTech Ltd London United Kingdom; 3 Xim Ltd Southamptom United Kingdom; 4 Faculty of Science & Health University of Portsmouth University Learning Centre Portsmouth United Kingdom; 5 National Institute for Health Research Barts Biomedical Research Centre London United Kingdom; 6 See Acknowledgments London United Kingdom

**Keywords:** general practice, vital signs/methods, vital signs/standards, photoplethysmography, remote photoplethysmography, rPPG, Lifelight, contactless, software

## Abstract

**Background:**

Measuring vital signs (VS) is an important aspect of clinical care but is time-consuming and requires multiple pieces of equipment and trained staff. Interest in the contactless measurement of VS has grown since the COVID-19 pandemic, including in nonclinical situations. Lifelight is an app being developed as a medical device for the contactless measurement of VS using remote photoplethysmography (rPPG) via the camera on smart devices. The VISION-D (Measurement of Vital Signs by Lifelight Software in Comparison to the Standard of Care—Development) and VISION-V (Validation) studies demonstrated the accuracy of Lifelight compared with standard-of-care measurement of blood pressure, pulse rate, and respiratory rate, supporting the certification of Lifelight as a class I Conformité Européenne (CE) medical device.

**Objective:**

To support further development of the Lifelight app, the observational VISION Multisite Development (VISION-MD) study is collecting high-quality data from a broad range of patients, including those with VS measurements outside the normal healthy range and patients who are critically ill.

**Methods:**

The study is recruiting adults (aged ≥16 years) who are inpatients (some critically ill), outpatients, and healthy volunteers, aiming to cover a broad range of normal and clinically relevant VS values; there are no exclusion criteria. High-resolution 60-second videos of the face are recorded by the Lifelight app while simultaneously measuring VS using standard-of-care methods (automated sphygmomanometer for blood pressure; finger clip sensor for pulse rate and oxygen saturation; manual counting of respiratory rate). Feedback from patients and nurses who use Lifelight is collected via a questionnaire. Data to estimate the cost-effectiveness of Lifelight compared with standard-of-care VS measurement are also being collected. A new method for rPPG signal processing is currently being developed, based on the identification of small areas of high-quality signals in each individual. Anticipated recruitment is 1950 participants, with the expectation that data from approximately 1700 will be used for software development. Data from 250 participants will be retained to test the performance of Lifelight against predefined performance targets.

**Results:**

Recruitment began in May 2021 but was hindered by the restrictions instigated during the COVID-19 pandemic. The development of data processing methodology is in progress. The data for analysis will become available from September 2022, and the algorithms will be refined continuously to improve clinical accuracy. The performance of Lifelight compared with that of the standard-of-care measurement of VS will then be tested. Recruitment will resume if further data are required. The analyses are expected to be completed in early 2023.

**Conclusions:**

This study will support the refinement of data collection and processing toward the development of a robust app that is suitable for routine clinical use.

**Trial Registration:**

ClinicalTrials.gov NCT04763746; https://clinicaltrials.gov/ct2/show/NCT04763746

**International Registered Report Identifier (IRRID):**

DERR1-10.2196/41533

## Introduction

The measurement of vital signs (VS) provides important information about a patient's health and, importantly, a change in VS may herald a deterioration in health [1]. Despite the importance of VS to inform clinical decision-making, the accuracy and timeliness of measurement are in need of improvement [2-4]. However, the measurement of VS requires using multiple pieces of equipment that need to be calibrated regularly and is time-consuming. It may also be uncomfortable and stressful for patients, potentially compromising the utility of the information obtained. Standard-of-care medical equipment is not suitable for patients who require regular measurement of VS in the home or community setting to monitor long-term health conditions because of cost, the complexity of the measuring processes, and the need for calibration of equipment. A study of 725 patients reported that while 53% followed at least 10 of the recommended steps necessary for accurate blood pressure (BP) measurement at home, only 1% followed all 15 recommendations [5]. Thus, home measurement of VS is important—and respiratory rate and pulse rate in particular—but requires several pieces of equipment (BP monitor and pulse oximeter) and for patients to be educated in best practices. The COVID-19 pandemic highlighted the need for remote or contactless VS measurement to reduce the risk of infection, which can be operated by people without specific medical training. The shift away from face-to-face to digital consultations during the pandemic also points to the need for easy but accurate measurement of VS.

Photoplethysmography (PPG) is an optical technique based on the measurement of the light reflected from the skin surface, which changes due to volumetric changes in the facial blood vessels; small variations in perfusion provide valuable information about the cardiovascular system [6]. PPG has been used to measure pulse rate [7,8], oxygen saturation [9], BP [10,11], and respiratory rate [7,12] and to detect atrial fibrillation [13].

Lifelight (Xim Ltd) is an app being developed for the contactless measurement of VS using remote PPG (rPPG) via the camera on smart devices such as phones and tablets. The app captures the average color of the region of interest 30 times every second for 60 seconds and sends this as red, green, and blue values to the server for further processing. VS values are obtained from the green channel.

The VISION-D (Measurement of Vital Signs by Lifelight Software in Comparison to the Standard of Care-Development) study measured VS in 8585 patients and healthy volunteers simultaneously using Lifelight and standard-of-care methods. The data were used for machine learning to improve the accuracy of the Lifelight algorithms used to calculate VS. The smaller VISION-V (Measurement of Vital Signs by Lifelight Software in Comparison to the Standard of Care–Validation) study demonstrated the accuracy of the Lifelight app compared with standard-of-care methods for measuring pulse rate, respiratory rate, and diastolic BP [14], providing the basis for the current class I Conformité Européenne (CE) registration [15]. However, some of the methods used in the VISION-V study differed from the procedures described in the standard for BP measurement (ISO81060-2) because of the novel nature of the Lifelight technology. Furthermore, these early studies did not include participants with BP values across the full range likely to be encountered in clinical practice.

To further improve the accuracy of Lifelight, the Measurement of Vital Signs by Lifelight Software in Comparison to the Standard of Care–Multisite Development (VISION-MD) study is collecting data from a wide range of outpatients, inpatients, and patients who are critically ill, and across the full range of skin tones, for use in machine learning. In VISION-D and VISION-V, full-face videos were recorded, but a high proportion of data were not usable. Thus, high-resolution full-face videos are being recorded to maximize the opportunity for machine learning. These data will also be used to evaluate alternative methods of defining the region of interest, as the full face includes areas that are not relevant (eg, areas covered by facial hair and areas that illicit a poor signal).

Given that only a small proportion of the raw video signal is relevant for rPPG measurement of VS (1%-2%), we are developing ways to enhance data collection and signal processing. Video recordings will be of higher resolution than those in the VISION-V and VISION-D studies, and data processing is focusing on the midface region (cheeks, nose, and top of the lip), rather than the whole face; these areas are computationally efficient for rPPG because of their large area and good-quality signal [16] but are not likely to be affected by autoregulation of cerebral blood flow (which discounts the forehead) [17]. We are also developing a method to identify small regions of interest in the midface in each participant where signal quality is the highest. This approach is expected to overcome some of the challenges of rPPG for routine clinical use, such as positioning of the participant relative to the light source.

VISION-MD (Clinicaltrials.gov NCT04763746) aims to advance the development and accuracy of the Lifelight app as a noninvasive and easy-to-use device to measure VS in hospitals and the community. The study will collect data from a broad range of patients to further develop the accuracy of Lifelight to a level sufficient for clinical applications, including screening and monitoring of cardiovascular disease. The initial data collected are being used for machine learning; later data will be used to test the accuracy of Lifelight compared with standard-of-care measurement of VS.

Thus, the primary objective of VISION-MD is to further develop the Lifelight algorithms across extensive clinical ranges, including critically ill patients and in patients with different skin tones. Secondary objectives are: (1) to improve and test the efficacy of Lifelight estimates for BP, pulse rate, respiratory rate, and oxygen saturation in multiple clinical settings (eg, critical care, outpatient clinics, and general hospital wards); (2) to evaluate the impact of variables on the accuracy of Lifelight VS measurements (eg, age, sex, temperature, health condition, medication, skin tone, and ambient lighting); (3) to understand the health economic potential of Lifelight; and (4) to compare the patients’ experience of current contact-based methods for measuring VS and Lifelight and to evaluate the patients’ acceptance and appetite for Lifelight.

## Methods

### Ethics Approval

The VISION-MD protocol was approved by the South Berkshire Research Ethics Committee on November 24, 2020 (20/SC/0432). Before the study started, the initial study protocol was approved by Health Research Authority (HRA) Wales on January 18, 2021 (IRAS 289242). HRA Wales has also approved subsequent protocol amendments.

### Participants and Recruitment

Participants are being recruited from multiple venues across Portsmouth Hospitals University NHS Trust, Barts Health NHS Trust, London, and from the community in London and Portsmouth (eg, religious places, community centers, offices, patient events, waiting areas in general practices, academic institutions, sports facilities, and care homes). The participants are inpatients, outpatients, friends and family of patients, visitors, hospital staff members, and the general public. The study staff approach inpatients during their hospital stay and outpatients while waiting for appointments. For adults lacking capacity (eg, critically ill patients), the next of kin are contacted by telephone. In addition, ethics-approved advertising materials are disseminated to Trust staff by email and in meetings, and posters are displayed in staff, patient, and public areas.

Inclusion criteria include individuals aged 16 years and older, sufficiently conversant in the English language, and able and willing to comply with all study requirements and to provide informed consent (either themselves or empowered by law to provide it). There are no exclusion criteria. Eligible potential participants are provided with an ethics-approved participant information sheet explaining the study aims, what is involved, and the requirements for participation; members of the team are available to discuss the study with interested individuals. Informed consent is obtained electronically using Research Electronic Data Capture (REDCap), a secure National Health Service (NHS)–compliant web-based platform for survey and database management (project-redcap-org). For adults lacking capacity, informed consent is obtained from a nominated consultee (next of kin or a doctor not involved in the study). Participation in the study is entirely voluntary, refusal to participate does not incur a penalty or loss of medical benefits, and participants may withdraw from the study at any time.

Recruitment started in May 2021 but was compromised by restrictions implemented during the COVID-19 pandemic to limit access to hospitals by the general public. Protocol amendments were made to increase community recruitment in light of these issues. Target recruitment is approximately 1950 participants to generate measurements for use in the initial training data set and for performance testing. However, the final sample size will depend on the incremental improvement in accuracy of the Lifelight algorithm and therefore cannot be predicted (see Sample Size section). The study will continue until the accuracy of Lifelight for measuring VS is sufficient for various clinical use cases.

### Study Procedures

#### Premeasurement observations

A brief set of demographic and medical history questions are asked, limited to the presence or absence of conditions that might affect skin perfusion and pigmentation and cardiovascular processes and any prescription medicines for these conditions. The study staff record a set of premeasurement observations and the presence or absence of sweat on the participant’s face; any facial hair on the cheeks; tattoos, jewelry, birthmarks, scars, or other features on the face; the use of foundation or concealer; and the position of the participant (seated, prone, supine, or lying on one side).

#### Subprotocol assignment

Patients with capacity are recruited into 1 of 3 subprotocols depending on premeasurement observations (Table 1). Participants may also be recruited to a subprotocol based on their skin tone (Fitzpatrick Skin Type scale [18]) to meet prespecified targets. Adults who lack capacity are recruited into subprotocol 4. Participants may be involved in up to 10 study sessions, allowing the collection of longitudinal data. The subprotocol approach allows the study personnel to focus on fewer tasks. It also enables high-quality data collection while avoiding the collection of data that would not be used to meet study objectives, consistent with the General Data Protection Regulation for data minimization.

**Table 1:** Recruitment criteria and vital sign measurement in subprotocols 1-4.

Subprotocol	Recruitment criteria	Measurements					Measurements, n
		PR^a^	BP^b^	RR^c^	SpO_2_^d^		
1	Abnormal BP^e^	✓	✓		✓	3	
2	Any participant			✓	✓	2	
3	Expected to have low SpO_2_^f^				✓	2	
4	Adults lacking capacity	✓	✓	✓	✓	3	

^a^PR: pulse rate.

^b^BP: blood pressure.

^c^RR: respiratory rate.

^d^SpO_2_: oxygen saturation.

^e^Abnormal defined as systolic blood pressure <100 mm Hg or >140 mm Hg.

^f^Low SpO_2_ (anticipated to be ≤95%).

#### VS measurement

The study staff ensure that participants have been at rest for at least 10 minutes before VS measurement starts and that they have not consumed any food or drink in the previous 30 minutes (other than intravenous fluids or nasogastric feeding). In each study session, VS is measured as per the subprotocol using the standard-of-care equipment while simultaneously capturing a video of the participant’s face using the Data Collect app running on a tablet (standard iPad 9.7, 2018) positioned approximately 1 m away and angled toward the participant’s face. Controls and instructions on the device start and stop the 60-second video recording. Background luminosity is measured using a handheld lux meter. The study staff have been briefed on the optimum Lifelight measurement conditions. Recordings are repeated once or twice, as set out in Table 1. The app does not return any measurements to the user or participant.

VS measurements are taken and coordinated by 2 nurses, one of whom announces the start and finish of the recording period on the Data Collect app. BP is measured using a standard clinical automatic sphygmomanometer with an appropriately sized cuff (width at least two-thirds of upper arm length) on the participant’s nondominant upper arm (unless contraindicated) or via an arterial line if fitted. BP is recorded at the start of the recording period. A standard clinical finger clip sensor for the measurement of oxygen saturation and pulse rate is placed on a finger on the opposite side of the body to the sphygmomanometer. Oxygen saturation and pulse rate are measured at 0, 30, and 60 seconds of the recording period and averaged. Respiratory rate is determined manually by counting chest rises throughout the 60-second period. The nurse may place their hand on the participant’s chest to increase the accuracy of manual counting but being mindful not to obscure the camera’s line of sight.

Each study session takes approximately 30 minutes. Once the measurements are completed, the study staff complete the postmeasurement observation questions relating to how much the participant moved, their position, whether they were wearing glasses, any hairstyle or other item (eg, face covering) that obscured any part of their face during the recording, and whether the software reported “face not found” at any point during the recording.

#### Patient Feedback

Equal proportions of participants in subprotocols 1-3 are being asked to complete a questionnaire related to VS measurement and their preferences. The data are fully anonymized and recorded without any identifiable information (including participant ID code).

#### Clinical Feedback

A questionnaire is available to garner feedback on the technology from the clinical user’s point of view (ie, the nurses who take the VS measurements). Questionnaire and interview data are fully anonymized and recorded without any identifiable information.

#### Health Economics Data Collection

The study also includes activities to obtain information and data to assess the cost-saving potential of Lifelight in different clinical settings, including as a tool to detect undiagnosed cardiovascular disease and to monitor symptoms. The cost of BP monitoring equipment and its maintenance and calibration will also be determined.

Stopwatch observational studies are run to determine how long it takes to measure VS using standard-of-care equipment and Lifelight, starting from the time when the clinician decides to conduct a VS check and incorporating the time it takes to find the measuring equipment, roll up the patient’s sleeve, put on the devices, wait for the result, and put the equipment away. This part of the study will involve approximately 20 participants.

### Privacy and Data Collection

Each study participant is assigned a unique sequential ID; no identifiable data are stored. All documents are stored securely and are only accessible by the study staff and authorized personnel. The code linking the ID to the participant’s personal information is kept within the hospital study site and can only be accessed by the research team.

Full-resolution video data are uploaded during the study. The consent form allows the participants to decide whether data can be shared as full-face video or with identifying features obscured.

Videos collected in the study constitute personal data, as it may be possible to identify participants, but are collected for research purposes only (not clinical care) and are processed within the legitimate interests of Xim Ltd. These data will be protected according to the General Data Protection Regulation.

### Data Handling

For each reading, a high-quality video of the whole face is saved to the internal storage of the iPad in encrypted form. Anonymized rPPG data (the average color of areas of the face) are saved directly and immediately sent to an NHS-compliant cloud server.

Subsequent analysis will be performed using the encrypted files, which are downloaded to a processing site, decrypted, and processed automatically (ie, without any person viewing the videos). This procedure will result in anonymized aggregate data sets. Decrypted files will subsequently be deleted from the processing site.

All protocol-required information besides video data is collected in an electronic case report form. The REDCap electronic cloud is used to store and manage all consent and study data. All data collected about study participants are kept strictly confidential.

### Performance Targets

The accuracy of Lifelight using the training data generated in VISION-D was sufficient to support the certification of Lifelight as a class I CE medical device [15]. However, the accuracy needs to be improved further for use in routine clinical practice. Table 2 lists the performance targets for Lifelight; training data collected during VISION-MD will support the progress toward these targets.

**Table 2:** Performance (accuracy) targets for Lifelight.

Vital signs	Accuracy target	Basis for target
Blood pressure	SBP^a^ can be measured with standard deviation ≤8 mm Hg British Hypertension Society Grade C for SBP measurement	ISO81060-2^b^ for blood pressure cuffs [19]
Pulse rate	Root mean square error of ≤3 beats per minute	Most common accuracy of CE^c^-marked commercially available devices
Respiratory rate	Maximum error tolerance of 5 breaths per minute	Accuracy of Philips Health watch, a CE-marked contact-based photoplethysmography device
Oxygen saturation	Maximum error tolerance of 4%	ISO80601-2-61 standard for pulse oximeters [20]

^a^SBP: systolic blood pressure.

^b^ISO: International Organization for Standardization.

^c^CE: Conformité Européenne.

### Sample Size

The sample size cannot be formally calculated because it depends on the incremental improvement in the accuracy of Lifelight achieved through machine learning using the training data generated in the study. However, indicative sample sizes for the 4 subprotocols have been calculated by assessing the optimal data requirements to enable algorithm training toward the standards defined in Table 3, balanced against the practicality of achieving the targets. The split between training and testing data will be determined during the study according to the quality of the data collected.

The initial protocol anticipated data collection from about 8400 participants for training and a further 1000 for independent testing of accuracy, but the recruitment has been compromised by restrictions implemented during the COVID-19 pandemic. However, the high-quality video recording (compared with VISION-D and VISION-V) supported a protocol amendment to reduce the recruitment to 1950 participants (see Results section), with the expectation that data from about 1700 will be used for training the algorithms and data from 250 used for testing. The study management team is monitoring the progress of data collection and accuracy, and updates the study teams monthly. The study will continue until the accuracy of Lifelight for measuring VS is sufficient for various clinical use cases.

As skin tone is expected to affect the accuracy of Lifelight, the aim is to recruit participants across the full Fitzpatrick skin tone scale (1-6). To allow the impact of skin tone measurement accuracy to be determined with statistical robustness, the full data set will be sampled to create a subset for skin tone analyses in which the prevalence of the usually less prevalent skin tones is amplified. This subset will contain 750-1000 measurements, with 15%-20% each from categories 1, 2 and 3, 4, and 5 and 6. These measurements should be spread across the subprotocols as indicated in Table 3.

**Table 3:** Indicative sample size targets.

Subprotocol	Indicative sample size, n^a^	Characteristics	Participants with skin tone categories 1, 4, 5, and 6
1	1500	Roughly 100 participants will be recruited with SOC^b^-determined SBP^c^ in each 10 mm Hg increment from <90 mm Hg to >200 mm Hg (ie, <90 mm Hg; 90-99 mm Hg; 100-109 mm Hg, etc)^d^	Ideally ≥4 in each SBP band
2	375	N/A^e^	Ideally ≥10 in each SBP band
3	35	Approximately 33% with SOC-measured oxygen saturation <88%, 88%-92%, and 93%-95%	Ideally, each band will include participants with each skin tone
4	No specific target; likely to be a small proportion	N/A	N/A

^a^Participants in subprotocol 4 (ie, those without the capacity to provide informed consent) are likely to have vital sign values outside of the normal range and will contribute to all subprotocol targets. Only the first study session per participant contributes to the sample size.

^b^SOC: standard of care.

^c^SBP: systolic blood pressure.

^d^Can include participants with SBP measured from an arterial line.

^e^N/A: not applicable.

### Data Analysis

The training data will be used to further develop the signal extraction and processing methodology. The test data will subsequently be used to determine the performance of Lifelight against the targets set out in Table 2.

All statistical analyses will be performed using Microsoft Excel. All analyses will be completed per protocol since there is no intention to treat.

There will be no imputation of missing or implausible data, and any missing, implausible, or problematic readings will be excluded from the analysis. If the Lifelight software is unable to detect the participant’s face during the measurement period, this will be recorded in the electronic case report form, and the measurements will be deleted from the data set.

## Results

The prototype Lifelight technology has been in development since 2016. The recruitment of participants for VISION-MD started in May 2021 but was compromised by the restrictions implemented to manage the COVID-19 pandemic, including restricting hospital access to the general public. Protocol amendments were thus made to enhance community recruitment, including the use of incentives such as chocolates or gift cards. In addition, the higher-resolution video recording (compared with the earlier VISION studies) supported reduction of the recruitment target to 1950, which is expected to yield sufficient high-quality measurements for machine learning and subsequent testing (reflected in a further protocol amendment). An additional amendment allowed the measurement of BP and pulse rate using devices other than the standard-of-care Welch Allyn devices (and indicated in the electronic case report form), as not all participating centers had the originally specified equipment.

Data for analysis will become available from September 2022, and the algorithms will be continuously refined to improve clinical accuracy. We anticipate that the final analyses to determine the performance of Lifelight against the targets set out in Table 2 will be complete in early 2023.

## Discussion

The VISION-MD study is expected to provide sufficient high-quality data from a wide range of healthy volunteers and patients (including critically ill patients) to further develop the accuracy of the software for estimating VS in clinical and community settings. While the VISION-V and -D studies demonstrated the potential value of Lifelight in the contactless measurement of VS and supported class I CE certification [15], further refinement of data collection and analysis methods is needed—particularly VS measurements outside the normal healthy range—to develop the algorithms for clinical use.

The high-quality videos collected in the VISION-MD studies will be instrumental in training the algorithms being developed for data processing. A proportion of the data collected will be retained for testing the performance of Lifelight in estimating VS compared with the standard of care.

The study findings will be published in high-impact peer-reviewed scientific journals and presented at international cardiology, respiratory, and medical device conferences.

## Abbreviations

BP: blood pressure
CE: Conformité Européenne
HRA: Health Research Authority
NHS: National Health Service
PPG: photoplethysmography
REDCap: Research Electronic Data Capture
rPPG: remote photoplethysmography
VS: vital signs
VISION-D: Measurement of Vital Signs by Lifelight Software in Comparison to the Standard of Care–Development
VISION-V: Measurement of Vital Signs by Lifelight Software in Comparison to the Standard of Care–Validation
VISION-MD: Measurement of Vital Signs by Lifelight Software in Comparison to the Standard of Care–Multisite Development

## Data Availability

The data generated and analyzed during this study are commercially sensitive and are therefore not publicly available, as mandated by Xim’s contractual obligations with its grant funders and investors. Furthermore, the informed consent provided by study participants only allows access to individual data, including in anonymized form, by authorized individuals of the research team based at the study sites, Xim, and Xim’s authorized partners. Reasonable requests for access to the study data within these limitations will be considered by the corresponding author.

## References

[1] Buist MD, Jarmolowski E, Burton PR, Bernard SA, Waxman BP, Anderson J (1999). Recognising clinical instability in hospital patients before cardiac arrest or unplanned admission to intensive care. A pilot study in a tertiary-care hospital. Med J Aust.

[2] Hands C, Reid E, Meredith P, Smith GB, Prytherch DR, Schmidt PE, Featherstone PI (2013). Patterns in the recording of vital signs and early warning scores: compliance with a clinical escalation protocol. BMJ Qual Saf.

[3] van Leuvan CH, Mitchell I (2008). Missed opportunities? An observational study of vital sign measurements. Crit Care Resusc.

[4] Ludikhuize J, Smorenburg SM, de Rooij SE, de Jonge E (2012). Identification of deteriorating patients on general wards; measurement of vital parameters and potential effectiveness of the Modified Early Warning Score. J Crit Care.

[5] Flacco ME, Manzoli L, Bucci M, Capasso L, Comparcini D, Simonetti V, Gualano MR, Nocciolini M, D'Amario C, Cicolini G (2015). Uneven accuracy of home blood pressure measurement: a multicentric survey. J Clin Hypertens (Greenwich).

[6] Kamal AA, Harness JB, Irving G, Mearns AJ (1989). Skin photoplethysmography—a review. Comput Methods Programs Biomed.

[7] Johansson A, Oberg PA, Sedin G (1999). Monitoring of heart and respiratory rates in newborn infants using a new photoplethysmographic technique. J Clin Monit Comput.

[8] Poh M, Poh YC (2017). Validation of a standalone smartphone application for measuring heart rate using imaging photoplethysmography. Telemed J E Health.

[9] Aoyagi T, Miyasaka K (2002). Pulse oximetry: its invention, contribution to medicine, and future tasks. Anesth Analg.

[10] Elgendi M, Fletcher R, Liang Y, Howard N, Lovell NH, Abbott D, Lim K, Ward R (2019). The use of photoplethysmography for assessing hypertension. NPJ Digit Med.

[11] Radha M, de Groot K, Rajani N, Wong CCP, Kobold N, Vos V, Fonseca P, Mastellos N, Wark PA, Velthoven N, Haakma R, Aarts RM (2019). Estimating blood pressure trends and the nocturnal dip from photoplethysmography. Physiol Meas.

[12] Nilsson L, Johansson A, Kalman S (2000). Monitoring of respiratory rate in postoperative care using a new photoplethysmographic technique. J Clin Monit Comput.

[13] Sun Y, Yang Y, Wu B, Huang P, Cheng S, Wu B, Chen C (2022). Contactless facial video recording with deep learning models for the detection of atrial fibrillation. Sci Rep.

[14] Heiden E, Jones T (2022). Measurement of vital signs using Lifelight® Remote Photoplethysmography: results of the VISION-D and VISION-V observational studies. JMIR Form Res.

[15] (2022). Medtech innovation briefing MIB213: Lifelight First for monitoring vital signs 2020. National Institute for Health and Care Excellence.

[16] Kwon S, Kim J, Lee D, Park K (2015). ROI analysis for remote photoplethysmography on facial video. Annu Int Conf IEEE Eng Med Biol Soc.

[17] Kashima H, Ikemura T, Hayashi N (2013). Regional differences in facial skin blood flow responses to the cold pressor and static handgrip tests. Eur J Appl Physiol.

[18] Colvonen PJ (2021). Response to: investigating sources of inaccuracy in wearable optical heart rate sensors. NPJ Digit Med.

[19] (2019). ISO 81060-2+A1: non-invasive sphygmomanometers— part 2: clinical investigation of intermittent automated measurement type. ISO.

[20] (2019). ISO 80601-2-61: medical electrical equipment— part 2-61: particular requirements for basic safety and essential performance of pulse oximeter equipment. ISO.

